# Developing ovarian cancer stem cell models: laying the pipeline from discovery to clinical intervention

**DOI:** 10.1186/1476-4598-13-262

**Published:** 2014-12-11

**Authors:** Brendan Ffrench, Claudia Gasch, John J O’Leary, Michael F Gallagher

**Affiliations:** Department of Histopathology, Trinity College Dublin, Trinity Centre for Health Sciences, St James Hospital, Dublin 8, Ireland; Pathology Department, Coombe Women’s and Infants University Hospital, Dublin 8, Ireland

**Keywords:** Ovarian cancer, Cancer stem cells, Stem cell models, Proliferate to kill, Clonal cancer stemness, Aldehyde dehydrogenase, Hoechst, Cluster of differentiation (CD) proteins

## Abstract

Despite decades of research, ovarian cancer is still associated with unacceptably high mortality rates, which must be addressed by novel therapeutic approaches. One avenue through which this may be achieved is targeting of tumor-initiating ‘Cancer Stem Cells’ (CSCs). CSCs are sufficient to generate primary and recurrent disease through extensive rounds of asymmetric division, which maintain the CSC pool while producing the tissues that form the bulk of the tumor. CSCs thrive in the harsh tumor niche, are generally refractory to therapeutic intervention and closely-linked to the Epithelial-Mesenchymal Transition process, which facilitates invasion and metastasis. While it is well-accepted that CSC-targeting must be assessed as a novel therapeutic avenue, few ovarian CSC models have been developed due to perceived and actual difficulties associated with the process of ‘CSC Discovery’. In this article we review contemporary approaches to CSC Discovery and argue that this process should start with an understanding of the specific challenges associated with clinical intervention, laying the pipeline backwards towards CSC Discovery. Such an approach would expedite the bridging of the gap between laboratory isolation and clinical targeting of ovarian CSCs.

## Introduction

Ovarian cancer develops silently towards presentation with advanced disease, which is generally successfully treated with a combination of surgical de-bulking and chemotherapy. In spite of initial treatment success, an unacceptably high number of patients (70%) develop terminal, recurrent, chemoresistant disease [1]. Clearly, patients require novel treatments that target the development of recurrent chemoresistant disease. One avenue through which this may be achieved is the targeting of ‘Cancer Stem Cells’ (CSCs), to which strong evidence points as the cell that is responsible for the development of chemoresistant recurrence.

CSC Theory states that only some cells from a heterogeneous tumor are capable of tumorigenesis. These cells have been collectively termed ‘CSCs’ due to their stem cell-like properties of self-renewal (SR), differentiation and tumorigenesis (malignant tissuegenesis). The definitive message from CSC Theory is that the development of CSC-targeting therapies would greatly improve current cancer treatments. However, the generation of CSC model systems from which such therapies may be developed is perceived to be unachievable except in specialized labs. Here, we review ovarian CSC research towards an argument that the development of CSC models is readily achievable, through the use of modern laboratory tools. Ultimately, we illustrate how CSC Discovery and validation can translate towards identification of mechanisms through which a treatment-resistant malignancy may be better targeted. As we will illustrate, this process should begin with an understanding of the specific clinical problems associated with ovarian cancer, from which a pipeline should be laid back towards CSC Discovery.

## Lessons from stem cell discovery

**Figure 1:**
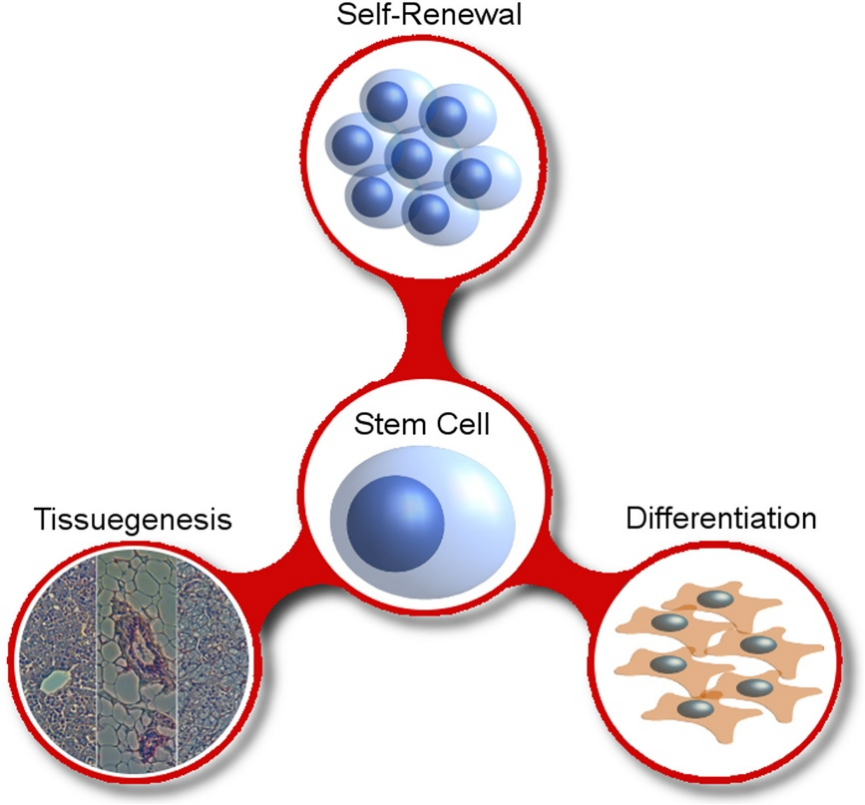
**The defining properties stem cells (SC) and cancer stem cells (CSCs).** SCs and CSCs can both be defined and validated via three properties. Self-renewal: (C)SCs can divide in a potency preserving fashion, producing two daughter stem cells. Differentiation: When necessary, (C)SCs can differentiate to produce daughter cells of reduced potency. Tissuegenesis: (C)SCs can derive the multiple cell types needed to form their given tissue. In the case of CSCs this is referred to as tumorigenesis (‘malignant tissuegenesis’).

Stem cells (SCs) are classified by their tissue of origin and their ‘potency’: the number of cell types they have the potential to generate. SCs are defined as cells capable of SR, differentiation and tissue-genesis (Figure 1). Through asymmetric division (AD; simultaneous production of undifferentiated and differentiated cells) SCs generate a cell hierarchy of high potency SCs, lower potency progenitors and terminally differentiated cells. Terminal differentiation is the end-point of stepwise differentiation. Terminally differentiated cells are responsible for the majority of tissue specialization and function. However, they no longer contribute to the generation of new cells.

**Figure 2:**
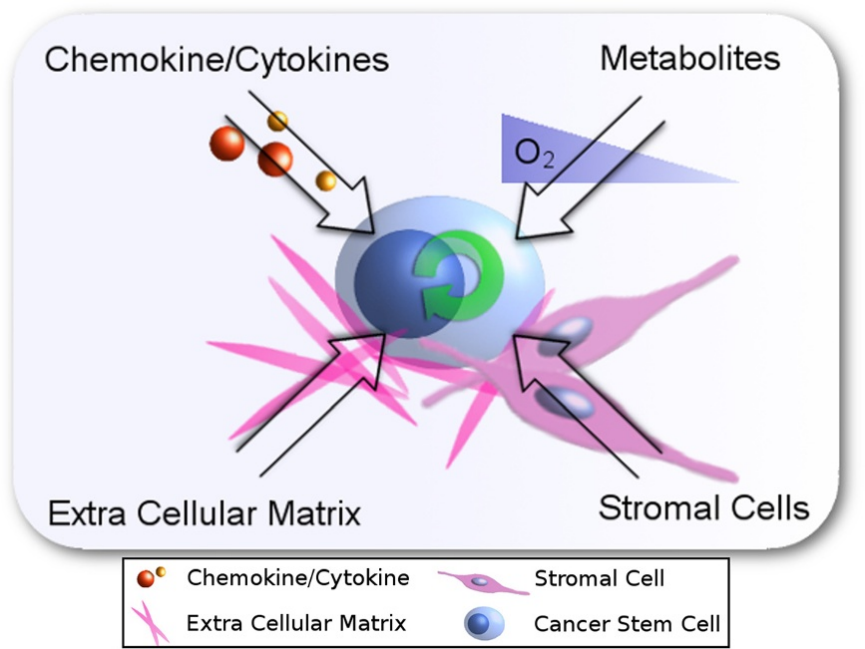
**The undifferentiated stem cell (SC) state is regulated by multiple factors in the stem cell niche.** Studies of the SC niche have shown that multiple factors regulate (C)SC activity. The most prominent factors range from chemokine/cytokine signaling and metabolite gradients to basement membrane and stromal cell interactions. Together, these factors regulate the undifferentiated state of SCs and CSCs.

As SCs were isolated from the body, it became clear that they required *in vivo* signals to maintain the SR state. In time, the SC micro-environment was shown to be influenced by cell to cell contact, autocrine and paracrine signaling proteins and environmental factors such as oxygen (Figure 2). At a molecular level, the mechanisms that maintain SR and facilitate differentiation are regulated by signaling pathways such as Hedgehog, Wnt, Notch and TGF-β. As CSC Discovery evolved, much progress was hastened through lessons from SC discovery, as it was found that aberrant regulation of SC mechanisms was responsible for malignancy.

## Cancer stem cell theory

It is now well-established that tumor-initiating cells from many, if not all, malignancies, share many properties with SCs, which has led to the collective term ‘CSC’. Today, CSCs are defined as being capable of SR, differentiation and generation of the original malignancy from which they were derived [2]. Historically, two fields of study ultimately converged to form the basis of modern CSC research. From their original recognition as embryonic-like tumors in the 1890s [3], single embryonal carcinoma (stem) cells were shown to be sufficient for tumorigenesis by 1964 [4] and had been developed into a pluripotent malignant SC model of SR, inducible differentiation and three-germ-layer tumorigenesis by the mid-1980s [5]. In parallel, substantial efforts to understand and treat leukemia in the decades following the 1945 nuclear attacks in Japan led to the description of leukemia SCs and the coining of the CSC term [6].

It is now understood that SCs and CSCs from the same tissue share many of the same SR and differentiation regulatory mechanisms [7]. While this complicates our ability to target CSCs in a manner that does not affect SCs, lessons learned from SC models can be exploited by CSC researchers. For example, following the discovery that Hematopoietic SCs (HSCs), and the myeloid and lymphoid progenitors and differentiated cells they produce (e.g. erythrocytes and leukocytes), were hierarchically organized, a similar hierarchy was demonstrated in leukemia [6].

The term ‘SC Hierarchy’ refers to the use of intermediate stem cells (referred to as ‘progenitor’ cells for clarity) in the production of tissues by SCs and CSCs. In recent years, Stem-Progenitor-Differentiated cell hierarchies have been described in many malignant and non-malignant tissues. In this model, the most powerful SC/CSC sits in a dormant ‘quiescent’ state at the apex of the hierarchy, from which it can be activated to produce progenitor cells (which produce differentiated cells) and then return to quiescence. In CSCs, such hierarchical organization can augment the tumors ability to overcome chemotherapeutic insults. For example, apex CSCs primarily reside in a stable quiescent state, outside of the cell cycle and thus are immune from anti-mitotic chemotherapies. Both SCs and CSCs utilize long periods of quiescence to protect against the stresses associated with cell division [8]. Entry to quiescence is regulated via p53-p21 signaling, allowing SCs/CSCs to exit the cycle to G_0_ from the G_1_ state. It has been recently reported that HSCs transition between G_0_ and ‘G_ALERT_’ states. This allows HSCs to rapidly return to the cell cycle, a process that appears to involve specific DNA-monitoring and repair mechanisms [9, 10].

As with SCs, CSCs states (SR, differentiation and quiescence) are determined by balanced suppression-activation of stemness signaling pathways such as Hedgehog, Wnt, Notch and TGF-β [11, 12]. A notable difference, with advantages for CSC discovery, is that CSCs do not spontaneously differentiate upon removal from the *in vivo* environment as readily as SCs. For example, the initially surprising discovery of CSC populations in long-established cancer cell lines is now common. While CSCs from these long-established cell lines may no longer be perfect facsimiles of the *in vivo* CSCs, they can be exploited for identification of cancer-specific CSC markers, which can be used subsequently to establish *de novo* primary CSC lines from tumor samples. As such, the traditional cancer cell line has become an easily-accessible starting point for CSC Discovery.

## CSC discovery overview

**Figure 3:**
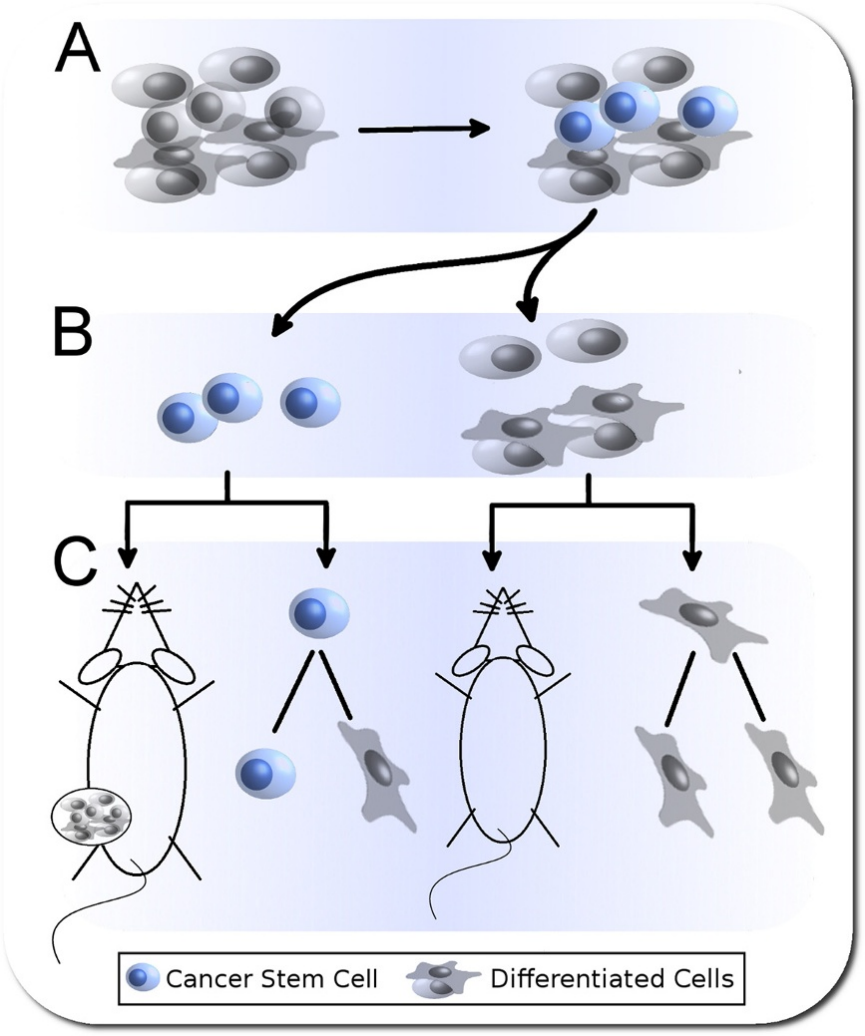
**Cancer Stem Cell (CSC) Discovery pipeline.** This figure illustrates the CSC discovery process. **A)** First, CSCs must be identified within a heterogeneous population of cells. As outlined in this review, CD133+, ALDH+ and HSP+ are the most successfully used ovarian CSC markers to date. Any CSCs identified must be considered putative CSCs (pCSCs) until they are validated as being capable of self-renewal, differentiation and the generation of a tumor with similar histology to that from which they were derived. **B)** Such pCSCs must be isolated to facilitate their validation. Fluorescence-activated and/or magnetic-activated cell sorting are commonly used to isolate pCSCs from non-pCSCs. **C)** Isolated cells can be validated by being assayed for tumorigenesis properties via xenograftment into mice, while their ability to self-renew and differentiate can be assayed via single cell asymmetric division assays.

As we will discuss in detail below, CSC Discovery spans from the identification of putative CSCs to their isolation and validation (Figure 3). Putative CSCs are identified by searching for cells that A) express so-called Stemness Markers and/or B) display stem cell behavioral properties. Stemness markers include Aldehyde dehydrogenase [13] as well as a large population of ‘Cluster of Differentiation’ (CD) proteins, of which CD44 is the most studied [14]. Stem cell behavior properties include the ability to efflux Hoechst dye [15] and to form spheroids in specific cell culture conditions. Developments in flow cytometry and the availability of suitable antibodies have enhanced our ability to identify and then isolate specific putative CSC populations from our cell line or tissue of interest. Subsequently, putative CSCs must be validated as true CSCs. This is commonly achieved by demonstrating efficient tumorigenic potential upon xenograft into immuno-compromised mice. Less popularly employed is the demonstration of differentiation potential via the Single Cell Asymmetric Division assay, which we will later argue is a very efficient CSC screen. Validated CSCs are then available for molecular analysis using standard techniques. Molecular data can subsequently be used to identify additional cell surface expressed proteins for more specific isolation of CSCs from appropriate tumor samples. Additionally, molecular analysis may highlight CSC mechanisms to which targeting strategies may be developed. However, as we will discuss in later sections, the most critical aspect of CSC Discovery is to ensure that the CSC source reflects the particular therapeutic challenges of the specific malignancy under study.

## The CSC is the culprit

The first step in the development of a CSC-targeting strategy is an understanding of how CSCs relate to the specific therapeutic challenges of the malignancy of interest. Ovarian cancer is infamous for high mortality rates due to advanced presentation and high levels of metastasis and chemoresistant recurrence. As such, ovarian CSC research must be directed towards early detection of the primary disease, chemoresistance and the development of the recurrent disease. Research from multiple malignancies has indicated that CSCs are associated with each of these processes, which has driven interest in developing ovarian CSC-targeting therapeutics. In particular, CSCs have been linked with ovarian cancer chemoresistance, which will be discussed later. To study their role in ovarian cancer disease progression, ovarian CSCs must first be identified, isolated and validated. While still limited by the lack of established ovarian CSC models, there has been much success in the identification, isolation and validation of ovarian CSCs, via CSC markers. These CSC markers are not necessarily important to the cancer stemness processes but may be utilized as disease and prognosis markers, as will be discussed later.

## Ovarian cancer has a strong track record with respect to CSC research

Epithelial ovarian cancers (EOC) represent ~90% of all ovarian cancer cases [16] and are thought to arise from ovarian surface epithelium stem cells [17] or fallopian tube epithelium [18]. As such, the majority of ovarian CSC research has focused on EOC. As we detail in this section, ovarian CSCs have been successfully identified based on the expression of stemness markers and/or stemness-associated biological properties. The collated reports are yet to culminate in description of a consensus CSC model for ovarian cancer. This likely reflects the complexity of ovarian tumorigenesis, which is likely to involve multiple, independent and/or interacting stem-progenitor-differentiated hierarchies [19]. Before therapeutic targets can be identified, this complexity must be elucidated, through the description of as many ovarian CSC models as possible.

### Isolation of ovarian CSCs based on aldehyde dehydrogenase expression

Stem cells commonly highly express the enzyme Aldehyde Dehydrogenase 1 (ALDH1), which has been exploited to identify and isolate CSCs from patient samples and cell lines representative of a range of malignancies including breast [20], colon [21], brain [22], liver [23], lung [24] and ovary [25]. ALDH1 is a cytoplasmically expressed enzyme central to the retinoic acid signaling pathway – a key pathway in stem cell differentiation. ALDH1 is responsible for the metabolism of retinal to its active form retinoic acid which can then enter the nucleus and regulate transcription via retinoic acid receptors [26]. ALDH1 detection has strong technical advantages as it is commercially available as a flow cytometry-based kit, which identifies SCs and CSCs based on their ability to metabolize a synthetic ALDH1 substrate to produce a fluorescent signal, a reaction inhibited by diethylamino-benzaldehyde, which can be exploited as a negative control. In systems with >80% ALDH+ sub-populations we have found that the inclusion of a 1:1 mix of known ALDH+:ALDH- cells is a useful control when setting the ALDH+ gate in the test sample, to avoid under estimation of true ALDH+ population size. ALDH detection can be combined with other SC markers to enhance analysis. In one such study, ALDH+/CD133+ cells were found to be more CSC-like than ALDH+ cells, which in turn were more stem like than ALDH- cells [25]. Additionally, ALDH+ fractions have been shown to enrich for or overlap with other ovarian CSC markers such as CD44 and CD133 [27–29]. ALDH screening has a strong track-record for identification of CSC sub-populations that were subsequently successfully validated (Table 1).

**Table 1:** **Identification and validation success rates of CSC markers in ovarian cancer**

Putative CSC marker	Putative CSCs identified	CSCs validated	Screened only (references)	Screened and validated (references)
CD133+	10 of 11	5 of 5	[25, 27, 29–31]	[28, 32–35]*
ALDH+	4 of 4	4 of 4		[25, 27, 28, 33]*
HSP+	4 of 4	4 of 4		[31, 36–38]
CD44+	12 of 12	1 of 2	[27–32, 36, 39]	[33, 40]
CD117+	7 of 11	1 of 2		[33, 40]
CD24+	7 of 7	1 of 1	[25, 28, 31, 32, 36]	[41]
CD24-	1 of 1	1 of 1		[30]
ABCG2+	2 of 3	0 of 2	[30]	[28, 38]

This table is ranked from top to bottom by the most frequently validated ovarian CSC markers. CSC validation is classified as a demonstration of increased xenograft tumorigenicity in the respective studies. Most studies screened for multiple CSC markers, while only bringing a sub-set forward for validation. This table makes the distinction between markers that were ‘screened only’ and those that were ‘screened and validated’ in the respective studies.

*[33] found that CD133+ and ALDH+ putative CSCs validated as CSCs in most but not all of the patients tested. In other patients there was no significant difference between CD133+ and CD133- or ALDH+ and ALDH- cells in tumorigenicity. Additionally, in a small sub-set of patients CD133- not CD133+ cells were identified as CSCs.

### Isolation of ovarian CSCs based on Hoechst Dye Efflux properties

Stem cell are commonly capable of excluding Hoechst 33342 dye due to high expression of drug efflux pumps (ABCB1, ABCC1-5, ABCG2) and active transport mechanisms [15], a property that is not shared by non-SCs. Originally highlighted for its ability to identify HSCs [42], the so-called Hoechst Side Population (HSP) assay has been successfully employed to identify and isolate CSCs from cell lines and patient samples, representative of several malignancies including brain [43], colon [44], lung [45], liver [46] and ovary [36, 37]. Although the HSP assay is not as popular as the ALDH assay, it offers similar advantages of commercial availability of a flow cytometry based assay with a negative control inhibitor ‘Verapamil’. Interestingly, ABCG2 expression has proved a far less successful marker for the identification of ovarian CSCs than the H342 exclusion technique (Table 1), suggesting a multi-component mechanism.

### Isolation of ovarian CSCs based on expression of cluster of differentiation proteins

The most popular choice of SC and CSC isolation markers are, by far, the ‘Cluster of Differentiation’ (CD) group of cell-surface expressed proteins. CD proteins were originally described for their association with various stages of hematopoietic differentiation, as established at human leukocyte differentiation antigen (HLDA) workshops [47]. CD proteins have the advantage of being exploitable for SC and CSC identification due to the large collection of commercially available fluorescently labeled antibodies. However, the CD approach requires prior knowledge of the cell-surface expression of the cell of interest or a potentially extensive and expensive screening approach. Additionally, SCs and CSCs are often only identified using two or more CD markers, which complicates their identification and analysis. Interestingly, despite their expression by SCs and CSCs, CDs are generally not functional requirements for (C)SC properties. The CDs of most interest to ovarian CSC research are now described.

### CD133+ ovarian CSCs

CD133 is a five transmembrane glycoprotein [48], high expression of which has been used to identify and isolate CSCs across several malignancies such as colon [49], prostate [50], liver [51], brain [52], pancreas [53] and ovary [54]. Although widely utilized as a CSC marker, little is known about the function of this protein. A growing body of evidence identifies CD133 expression as marking a CSC component within ovarian cancer (Table 1). Some studies suggest that CD133 expression is among the most robust methods of identifying ovarian CSCs from patient tumors [32, 33], while others suggest that a ALDH+/CD133+ combination identifies a more stem-like population within ovarian cancer [25]. It has also been suggested that CD133+ cancer cells may be capable of trans-differentiating into endothelial-like cells to facilitate angiogenesis [55]. This points towards an ovarian cancer CD133+ population that is not intrinsically more tumorigenic but rather facilitates angiogenesis within the xenograft tumors, which augments the malignant potential of the cell inoculum.

### CD24+ ovarian CSCs

CD24 is a cell membrane protein originally described for its association with the differentiation of B-lymphocytes [56]. CD24 has been used to identify CSCs from a small number of malignancies and its expression has been both positively and negatively correlated with cancer stemness. For example, a CD44+/CD24+/ESA+ phenotype has been used to identify pancreatic CSCs [57], while a CD44+/CD24-/Lineage- phenotype has been used to identify breast CSCs [58]. The ambiguity of this putative CSC marker also extends to the ovarian CSC field (Table 1). CD24+ cells have been validated in mice studies as being more stem like than CD24- cells isolated from clones generated from the tumor cells of an ovarian cancer patient [41], while CD24- cells have been validated in mice studies as being more stem like than CD24+ cells and unsorted cells isolated from an ovarian cancer cell line [30]. This complexity reflects the main disadvantage of the CD approach.

### CD44+ and CD117+ ovarian CSCs

CD44 is the best known of the CD CSC markers but is generally an uninformative and ill-advised choice as a single marker as it does not appear to discriminate between the multiple stem and progenitor cells present in most tissues and malignancies (Table 1). However, when combined with other markers, CD44 has been successfully employed to identify SCs and CSCs from many tissues and malignancies. Here, we will describe the CD44+/CD117+ combination, which has been shown to mark ovarian CSCs [40]. CD44 and CD117 appear to have unrelated biological functions. Primarily, CD44 is considered a receptor for hyaluronic acid [59] and has been linked to a large number of cellular and tissue functions from cell migration [51] to cell proliferation [60] and cytokine, chemokine regulation [61]. CD117 is a type III receptor tyrosine kinase [62] that is primarily known as a receptor for Stem Cell Factor (SCF) and is proposed to have anti-apoptotic effects [63]. In addition to ovarian CSCs, CD44+ marks prostate CSCs [50, 64], while CD117+ marks CSCs in leukemia [65] and osteosarcoma [66]. As few as 100 CD44+/CD117+ ovarian CSCs are sufficient for *in vivo* tumorigenesis [40]. However, the phenotype is disputed as an ovarian CSC marker in a similar fashion to CD24. For example, it has been reported that CD44+/CD117+ cells are only detected in ~30% of serous ovarian carcinomas and that these cells did not augment tumorigenicity [33]. Again, the complexity of multiple stem and progenitor cells is most likely to be responsible for this contradiction.

### Isolation of ovarian CSCs based on spheroid-formation properties

It is now well-established that SCs and CSCs form spheroids when grown in anchorage-independent conditions, which were originally described as optimal for the *in vitro* propagation of normal neural progenitor cells in an ‘undifferentiated’ state [40, 67–69]. It is believed that these ‘Spheroid-Formation’ conditions are a method of CSC enrichment, rather than isolation, as there is no physical separation of the CSC and non-CSC components from the heterogeneous population. Instead, spheroid-formation appears to select for dominant SR of the CSC population, which proliferates to produce a larger CSC population. For example, it has been demonstrated that only cells that formed spheroids in these conditions, and not the cells which remained adherent, could be returned to exponential growth under normal culture conditions and then back to spheroid conditions to reform spheroids [69]. This would suggest that spheroid growth is a selective, rather than a transformative process. This approach has been advanced by commercial availability of several stem cell media products, which promote anchorage-independent growth based on a low percentage of serum and combinations of growth factors such as leukemia inhibitory factor (LIF), fibroblast growth factor (FGF), epithelial growth factor (EGF) and insulin. Spheroid growth has been primarily used as a popular assay to validate SC properties in putative CSCs (neural [53, 67], mammary [70] and melanoma [69]) and has been shown to be augmented in ovarian CSC populations [40]. Numerous publications have now validated spheroid growth, via xenograft tumorigenesis, as a method of enriching for ovarian CSCs relative to control cells grown in adherent conditions [30, 65, 71, 72].

The Spheroid-Formation approach has the major advantage of not requiring any prior knowledge of CSC markers expressed on the cell of interest. However, it has three main disadvantages; A) Downstream comparisons, including xenograft validations, of CSCs and non-CSCs often have to be made between cells propagated in different culture conditions (spheroid versus adherent growth); B) The purity of the ‘isolated’ CSC population cannot be quantified; C) Downstream mechanistic and functional analysis such as transfections and drug treatments are difficult to perform in spheroid formation conditions. These limitations reduce the power to identify therapeutically targetable pathways upon characterization.

### Isolation of ovarian CSCs based on holoclone-formation properties

Specialized agar based culture conditions or single cell plating can produce holoclones, meroclones and paraclones. These are considered to be clones produced from cells of reducing differentiation potential, ranked from high in holoclones to low in paraclones [73, 74]. In theory, this allows for downstream comparison of CSCs to non-CSCs.

In both a pancreatic and prostate cancer context, holoclone formation has been shown to enrich for CSCs relative to meroclones and paraclones [73, 74]. Interestingly, the holoclones were not shown to be more tumorigenic than the unselected parent cell line [73, 74]. To date the holoclone formation method has not been validated as a method for the enrichment of ovarian CSCs.

### Ovarian CSC isolation approaches in summary

To date there are no definitive ovarian CSC markers which identify ovarian CSCs across all populations tested. There is no definitive map as to how each of the validated ovarian CSC markers (ALDH+/CD133+: [25]; HSP+: [36]; CD133+: [32]; CD44+/CD117+: [40]) relate to each other. However, such a map is starting to materialize, with the expression of an as of yet unidentified marker(s) identifying the apex CSC in a complex ovarian CSC hierarchy [19]. Ovarian cancer is believed to be derived from ovarian surface epithelium SCs [17] or fallopian tube epithelium [18]. At first, it may appear counter-intuitive that there should be such CSC diversity originating from such simple epithelial sources. However, the developmental origins of the ovarian surface epithelium and the fallopian tube epithelium may have a role to play in the spectrum of cell lineages (serous, endometrioid, mucinous, clear cell) observed within ovarian cancer [75, 76].

## Validating CSCs via single cell asymmetric division and xenograft assays

**Figure 4:**
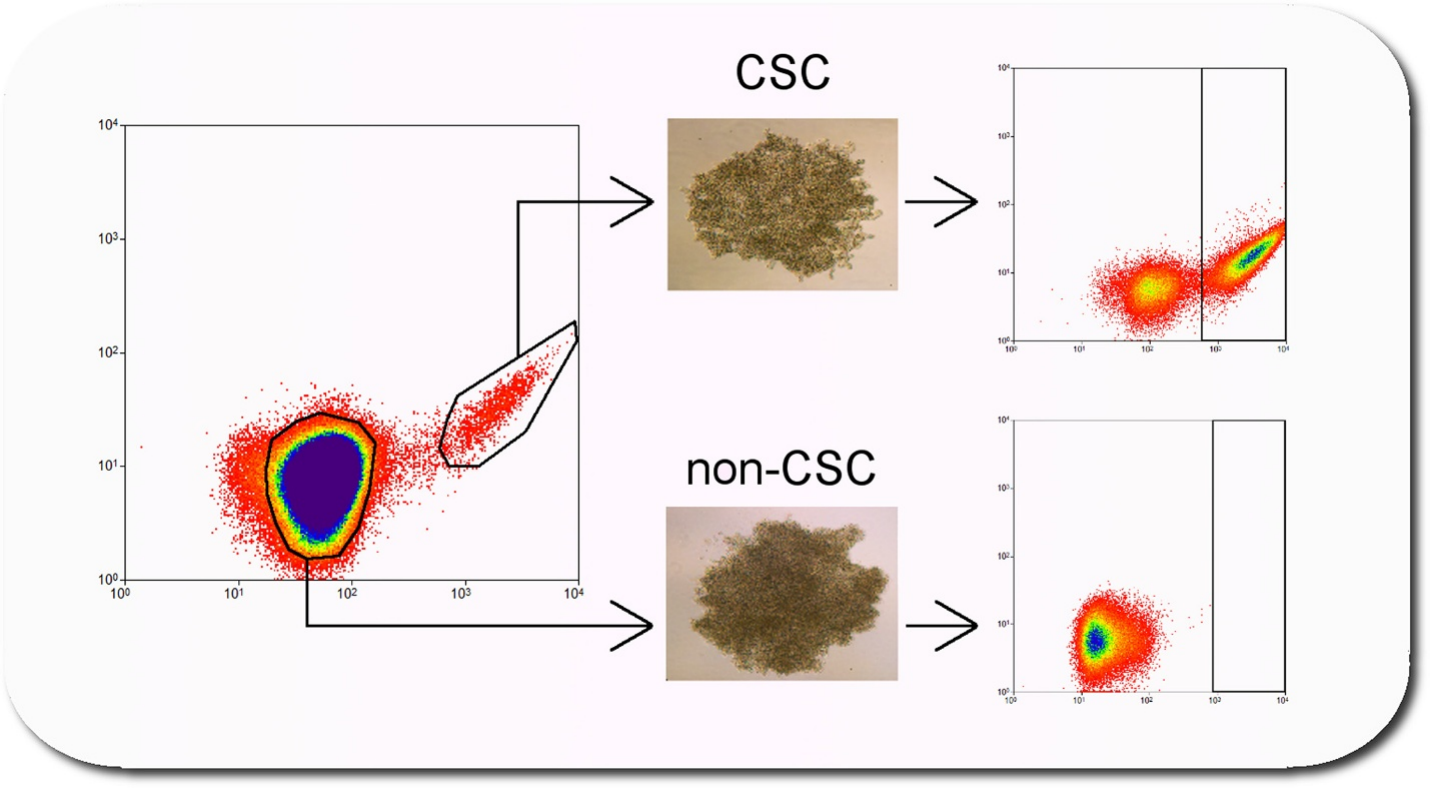
**The single cell asymmetric division assay (SCAD assay).** Utilizing cancer stem cell (CSC) markers via fluorescence-activated cell sorting, putative (pCSCs) and non-pCSCs are plated as a single cell per well in a 96-well plate. Such cells are allowed to form colonies, which are cultured to generate sufficient cells to re-analyze for the presence of the original CSC markers. If a clone reconstitutes the parent phenotype, it has demonstrated the CSC-like capacity to self-renew and differentiate. If a clone fails to grow or does not reconstitute the parent phenotype, it has demonstrated a reduced differentiation potential characteristic of non-CSCs. The SCAD assay used in parallel with the xenograft tumorigenicity assay can efficiently validate CSCs and non-CSCs.

Currently, validation of CSCs requires demonstration that low numbers of the putative CSC population are sufficient to regenerate the original malignancy following xenograft into immune-compromised mice. Xenograft tumorigenicity assays demonstrate the malignant potential of the CSCs and their ability to differentiate to produce the histology of the original tumor from which they were derived. Serial xenograftment of CSCs demonstrates the SR of such CSCs but this is not universally performed as it can be technically challenging and doubles the duration and expense of *in vivo* studies. In our experience, single-cell asymmetric division (SR and differentiation) assays (Figure 4) are less technically challenging and can run concurrently with *in vivo* validations. This streamlines progression from identification of CSCs to development of stable models. This assay involves the generation of cultures from individual, single-plated putative CSCs of known phenotype. These cultures can subsequently be screened for the presence of the original phenotype and/or new phenotypes, which indicates an ability to asymmetrically divide. Single cell clonogenicity assays have been used in the validation ovarian CSCs from cell lines [34] but its utility in testing putative CSCs from patient samples is unclear. The combination of these assays can validate putative CSCs as true CSCs for further analysis.

## CSC-associated proteins show promise as diagnostic and prognostic bio-markers

Ovarian CSC research has produced a promising panel of CSC markers. These markers have not been functionally linked with ovarian CSC properties such as SR and differentiation. For example; while ALDH1 is recognized as a marker of CSCs it is not necessarily required for the stemness characteristics of CSCs. This point is illustrated by the finding that *in vivo* siRNA knockdown of ALDH1 in ALDH+ cell lines showed no significant inhibition of tumorigenesis in a xenograft mouse model [47]. As it stands, CSC markers do not appear to be good candidates for CSC-targeting. However, their use as bio-markers is potentially very good but currently very poor. In response to identification of specific markers associated with different disease types, breast cancer patients are now being given the appropriate treatment regime and avoiding inappropriate regimes, which has dramatically improved survival rates. The combination of CSC and non-CSC bio-markers may permit similar improvements in ovarian cancer. For example, we and others have already identified Toll-Like Receptor Signaling pathway modulator MyD88 as a CSC-regulator that is associated with poor prognosis in ovarian cancer patient samples [77–79]. We have previously identified mitotic spindle checkpoint component MAD2 as another prognostic indicator [80]. It is possible that the combination of MyD88, MAD2 and other ovarian cancer bio-markers can permit patient-specific treatments to be developed.

Likewise, CD markers do not only facilitate identification and isolation of CSCs but have broader diagnostic and prognostic implications. For example, both ALDH1 and CD133 expression have been linked to poor prognosis in ovarian cancer [81–83]. One study generated a gene expression signature by examining data for 100 genes in 41 established ovarian cancer cell lines [34]. This gene expression signature was used to successfully segregate ovarian patients with tumors containing CD133+ cells from patients with completely CD133- tumors. The false positive rate of this segregation was 7.1% (3 in 42), while the false negative rate was 4.7% (2 in 42). Such gene signature approaches to the identification of CSC populations could prove especially powerful in the triaging of ovarian cancer patients.

## CSCs survive therapy and spawn the recurrent chemoresistant Phenotype

CSCs are linked to the most aggressive and lethal traits of malignancy. Single CSCs are sufficient to initiate and drive primary and recurrent disease [4, 58]. There is strong evidence linking CSC mechanisms to Epithelial-Mesenchymal Transition (EMT), which is a functional requirement for invasion and metastasis [84]. CSCs have also been linked with chemoresistance via detoxification, drug efflux and quiescence. CSC-targeting is clearly an attractive avenue through which novel ovarian cancer treatments could be developed. Interestingly, several studies to date have shown that CSC targeting alone is less efficient than targeting CSCs and treating with chemotherapeutic agents in combination [85, 86]. For example, Notch Signaling inhibition has been shown to dramatically decrease metastasis in a mouse model of ovarian cancer, but only when combined with cisplatin [86].

It is well-established that CSCs are chemoresistant [87]. For example; ALDH+ ovarian CSCs have been consistently shown to exhibit increased chemoresistance [25, 29, 82], with the size of the ALDH+ sub-population often correlating with acquired taxane and platinum resistance [26, 82]. Interestingly, xenograft mice studies have shown that treatment with chemotherapy drug cisplatin increases the size of the ALDH+ tumor sub-population [82], suggesting that ALDH+ cells have a selective advantage under chemotherapeutic conditions. Furthermore, siRNA mediated *in vivo* suppression of ALDH1 in xenograft tumors demonstrated a significantly (p < 0.013) improved response to platinum and taxane therapies [27]. Taken together, these data suggest that not only do ALDH+ cells have a selective advantage over the rest of the tumor but also that ALDH1 plays a central role in chemoresistance and likely contributes to the ALDH+ CSCs’ selective advantage. Similar to ALDH+ CSCs, HSP+ CSCs have also been shown to contribute to the chemoresistant phenotype in ovarian cancer. HSP+ ovarian CSCs have demonstrated augmented chemoresistance against cisplatin and doxorubicin but not paclitaxel [37, 49]. Verapamil treatment was able to reverse the chemoresistance to cisplatin suggesting drug efflux as the mechanism of chemoresistance in HSP+ cells [37].

Some of the molecular mechanisms behind recurrent ovarian chemoresistance are understood. For example, increased DNA repair/p53 mutation status, as well as elevated glutathione and metallothionein levels, is associated with platinum resistance [88, 89]. Taxol resistance has been found to be p53 independent and is associated with alterations in β-tubulin isoforms [90], which has recently been linked to the expression of the ovarian CSC marker CD44 [91]. In concordance with this, CSCs are known to have a highly regulated p53-p21-Rb pathway, which permits cell cycle entry-exit during periods of quiescence, rapid proliferation and differentiation [8, 92]. We have previously demonstrated that primary and recurrent serous papillary ovarian cancer patient samples differentially express thousands of genes [93, 94]. Notably, we subsequently showed that CSC mRNA and microRNA signatures were prominently expressed in ovarian tumor samples and differentially expressed by these primary and recurrent tumors [95, 96]. Considering all the evidence, it would appear that the small population of cells that survive the initial treatment either have a different expression profile or adapt their expression profile upon recurrence to generate a new tumor mass that can allow them to tolerate second-line chemotherapeutics. CSCs are a likely candidate cell for the generation of recurrence and as such are an attractive therapeutic target.

## Treating chemoresistance by targeting the appropriate CSC and its parent

Despite decades of research an effective method of treating recurrent chemoresistant ovarian disease has not been developed. One way to view this problem is that it represents our failure to identify and target the CSCs responsible for chemoresistant disease. To date, it has not been definitively demonstrated whether CSCs are inherently chemoresistant or adapt during treatments to develop chemoresistance. Additionally, it is not known whether all or only specific CSCs in the hierarchy are responsible for chemoresistance. For example, chemoresistance may be the property of the apex CSC or progenitor CSCs or both. Therefore, once a CSC hierarchy has been identified, it is important to highlight the specific CSC sub-population(s) responsible for chemoresistance rather than tumorigenesis per se.

As such, it is important to identify, study and target the specific ‘Chemoresistance Driving CSCs’. When considered in the context of the hierarchical organization of CSCs, chemoresistance driving CSCs must be characterized as either apex CSCs or progenitor CSCs. Where these are apex CSCs a direct target strategy is appropriate. However, where these are found to be progenitor CSCs, the apex CSC that produces ‘chemoresistance driving CSCs’ must also be identified, studied and targeted. By targeting both cell types simultaneously, treatments would ensure the removal of the CSC/progenitor responsible for chemoresistant disease and the apex CSC responsible for their replacement. Thus, models selected at the outset should be reflective of precise aspects of the malignancy of interest, as discussed below.

## ‘Clonal cancer stemness’ – the model predicting poor clinical outcome

**Figure 5:**
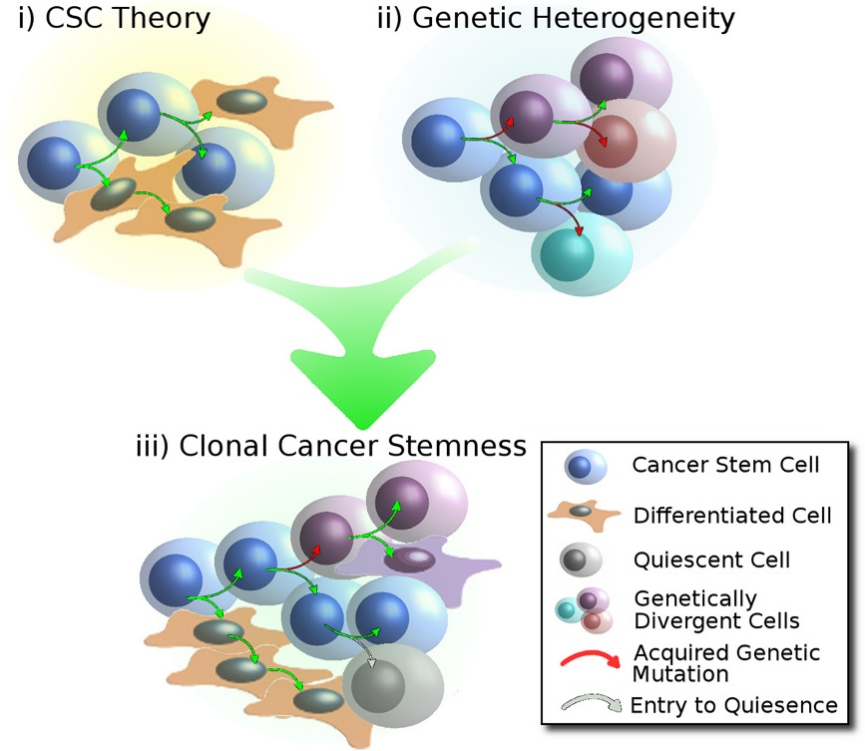
**Clonal Cancer Stemness. i)** CSC Theory states that tumors are composed of multiple cell types produced by CSCs via multiple rounds of self-renewal and differentiation. **ii)** The Genetic Heterogeneity viewpoint states that cumulative acquired genetic mutations lead to genetically divergent cell lineages. CSC Theory and Genetic Heterogeneity are not mutually exclusive concepts. In fact, growing evidence suggests both are correct. **iii)** This points towards the Clonal Cancer Stemness model of malignancy, where both CSCs and genetic divergence account for the heterogeneous traits of malignancy (e.g. chemoresistance and metastasis). Additionally, CSC hierarchical arrangement allows for CSCs to enter periods of quiescence, which can complicate current therapeutic approaches.

As discussed above, ovarian cancer chemoresistance can be attributed to intrinsic and adaptive resistance of CSCs as well as divergent genetic mutations. By combining both models of genetic heterogeneity and cancer stemness, we arrive at a model of ‘Clonal Cancer Stemness’ (Figure 5). A Clonal Cancer Stemness model of ovarian cancer predicts the failure of unilateral therapeutic approaches. Anti-mitotic therapies, such as treatment with platinum and taxane, have multiple single point failures predicted by the Clonal Cancer Stemness model: 1) A genetically divergent clonal lineage of cells with acquired chemoresistance can survive chemotherapy and reconstitute the malignancy with a dominant chemoresistant phenotype. 2) Intrinsically resistant CSCs may survive chemotherapy, adapt to the environmental insult and regenerate chemoresistant disease. 3) Similarly, a quiescent CSC population may also survive and adapt to generate recurrent chemoresistant disease.

Alternative approaches are required to overcome the limitations of unilateral anti-mitotic therapies. Ovarian cancer is uniquely positioned to take advantage of “weaponized” sex determining growth factors in the treatment of cancer. Müllerian inhibiting substance (MIS), a growth factor which inhibits the growth of female reproductive organs in male embryos, showed inhibitory effects on the proliferation of HSP+ CSCs [49]. Similar MIS induced inhibitory effects have also been demonstrated with CD44+/CD24+/EpCAM+ ovarian CSCs [97]. Such novel therapeutic approaches used in combination with contemporary anti-proliferation approaches should yield more success in the treatment of ovarian cancer, as there is a lower probability of a clonal lineage being resistant to both therapeutic approaches. Indeed, strong evidence is emerging that CSC-targeting in conjunction with anti-mitotic therapeutics is beneficial in otherwise refractory disease. This has been investigated using γ–secretase inhibitors (GSIs), which block Notch Signaling, a key pathway for CSCs and metastasis (EMT). In one example, the metastasis of pancreatic disease to the liver, which is otherwise gemcitabine-resistant, was dramatically reduced by treatment with gemcitabine and GSI combinations [85].

Clearly, deploying anti-mitotic therapies alongside anti-CSC targeting has potential to succeed where our current strategies fail. However, even such a bi-lateral approach has a single point failure predicted by the Clonal Cancer Stemness model. CSCs often reside in quiescent states, which equips them with intrinsic resistance to anti-mitotic and most likely anti-CSC therapeutics. New therapeutic approaches may have to overcome quiescence prior to utilizing anti-mitotic and anti-CSC approaches.

## ‘Proliferate to kill’ - awakening the sleeping CSC

Contemporary CSC Theory indicates that CSCs rapidly proliferate during early tumorigenesis, after which CSCs at the apex of the hierarchy move in to an inactive ‘quiescent’ state (Figure 5). Subsequently, tumorigenesis appears to be driven by (progenitor) CSCs lower down the hierarchy. In this quiescent state the apex CSCs reside in G_0_, outside of the cell cycle and immune to the effects of anti-mitotic chemotherapeutics. In this model, the treatment of primary disease by surgical de-bulking and chemotherapy, which is standard in ovarian cancer, would result in an awakening of the quiescent CSC to re-initiate tumorigenesis. As evidence for this, paclitaxel treatment and partial surgical de-bulking have been shown to augment the tumor growth of CD44+/MyD88+ ovarian CSCs in xenograft mice models via the pro-inflammatory TLR2-MyD88-NFκB pathway [98]. It appears that removal of active CSCs stimulates quiescent CSCs into activity, which could explain recurrence.

To address this challenge, ideally both the active and quiescent CSC populations should be targeted. However, quiescent cells are by definition difficult to identify, isolate and study. An alternative for future treatments is a ‘Proliferate to Kill’ strategy, which aims to stimulate the quiescent CSC population, forcing them to re-enter the cell cycle, thus exposing them to standard chemotherapy. A parallel strategy is being promoted in circumventing the leaky vasculature found in tumors, which hinders delivery of chemotherapy agents to all parts of the tumor. In this strategy, vasculogenesis is initially promoted by pro-VEGF drug-treatment, after which chemotherapy can be more effectively delivered to more tumor regions [99]. It may be necessary to induce proliferation of quiescent CSCs prior to targeted therapy. It has been demonstrated in mice that a single dose of 5-fluorouracil (5-FU; 1.5 mg/10 g body weight) could deplete both the myeloid and lymphoid compartments of the blood [100]. However, the blood count started to recover at ~ 8 days and was normal by 15 days. In contrast, mice treated with a second dose 3 or 5 days after the first showed a 75% and 86% reduction in reconstitution respectively. This reduction was not observed if the second dose was at day 1 or 8 after the initial dose [100]. This suggests that the quiescent HSCs are resistant to the 5-FU, but become sensitive when induced to proliferate to replenish the myeloid and lymphoid compartments. Proliferate to Kill presents an obvious caveat with particular relevance to ovarian cancer, as it proposes enhancing tumorigenesis in women already presenting with advanced disease. As such, extensive research will be required to assess the feasibility of adding the Proliferate to Kill strategy to existing ovarian cancer treatments.

In an ovarian cancer context, a two stage approach – first: induced hyper-proliferation, second: anti-proliferation and forced differentiation/‘weaponized’ growth factors – should be more successful than current unilateral anti-mitotic approaches. Before such strategies can be deployed, we first need to generate appropriate models of CSCs, through which we can investigate and validate novel therapeutic approaches.

## The absence of stable models of ovarian CSCs is a hindrance to the Identification of therapeutic targets

Stable CSC culture models are those that remain in the SR state without spontaneous differentiation. Stable models facilitate controlled differentiation via addition/removal of a growth factor or morphogen and tolerate transfections, drug treatments etc. As no stable models currently exist, ovarian CSC models need to be developed to facilitate their study. Isolated ovarian CSCs populations are unstable under normal culture conditions and quickly revert back to a heterogeneous population of CSCs and non-CSCs [49, 89]. Currently, the major obstacle to developing stable ovarian CSC models is defining the culture conditions necessary for the maintenance of the SR state of ovarian CSCs. Following on from that, the tissue source of ovarian CSCs needs to be carefully considered so that it correctly informs the malignant trait under investigation.

### Maintaining the SR state of ovarian CSCs

Current evidence is pointing towards spheroid culture conditions (LIF, FGF, EGF and Insulin) as being able to maintain the stem-like state of isolated ovarian CSCs [29, 39]. While this is a step in the right direction, much is yet unknown about the factors involved in the spheroid culture induced ovarian CSC self-renewal. Additionally, a stable ovarian CSC model must be grown in anchorage-dependent conditions to better facilitate cell culture analysis of mechanism and function via transfection of siRNAs, drug treatments etc. As such, the specific growth factors required to maintain ovarian CSCs in an undifferentiated state in culture must be identified. For example, this approach has facilitated a better understanding of Neural Stem Cell (NSC) SR. Spheroid culture was first developed for the culture of NSCs [68]. It has been shown that EGF acts through the Akt pathway to phosphorylate Bmi-1, which leads to its nuclear accumulation and increased proliferation of NSCs [101]. Additionally, FGF has been shown to promote the survival and/or undifferentiated state of NSCs where IGF-1 and EGF alone cannot [102]. Experiments in fibroblast proliferation suggest that IGF-1 signaling augments EGFR signaling by optimizing EGFR sub-cellular localization [103]. Further research is needed to investigate if spheroid culture can identify the growth factors necessary for maintaining the ‘undifferentiated’ state of ovarian CSCs and to develop techniques to uniformly force-differentiate ovarian CSCs. An alternative approach, studying the CSC-niche within the tumor microenvironment, has also shown success in identifying IL-17 as an important cytokine in the maintenance of SR in ovarian CSCs. It was demonstrated that IL-17 treatment increased the spheroid formation capacity and tumorigenic potential of ovarian CSCs, an effect linked to p38, MAPK and NFκB activation [104].

Isolating CSCs from the most appropriate source is of equal importance to the maintenance of SR in CSCs in the establishment of CSC models. Metastasis and recurrent chemoresistant disease are the major therapeutic challenges with respect to curing ovarian cancer patients. As will now be discussed, the isolation source of CSCs is of critical importance when establishing models to address these challenges.

### CSC sources define the utility of any ovarian CSC model established

Cell lines are a cornerstone of cancer research. The ubiquitous presence of CSCs in established cell lines makes them an obvious starting point for understanding the basic biology of ovarian CSCs. From there, work can be progressed into patient samples, allowing for identification, isolation and establishment of primary CSC cultures. Careful consideration should be given to the disease stage of patient material used. For example; CSCs isolated from a treatment naïve tumor and later a patient matched recurrent chemoresistant tumor would enable the study of CSC chemo-adaptation in the clinical setting. Furthermore, the CSC model established from the naïve state can be challenged with chemotherapeutics *in vitro* or *in vivo* via xenograft models and compared at the molecular level (gene sequencing, expression and methylation) to the pair matched CSC model established from recurrent chemoresistant patient tumor. This effectively generates a model system through which treatment strategies can be assessed to circumvent the acquisition of CSC driven chemoresistance in a clinical setting.

Another consideration is the source of the cancerous tissue within the patient. Approximately 40% of ovarian cancer patients develop ascites [105], where ovarian cancer cells shed into the peritoneal cavity and block the lymphatic drainage of the peritoneal fluid [106]. This, in conjunction with increased peritoneal fluid production via the tumors’ leaky vasculature, results in the build up of peritoneal fluid laden with ovarian cancer cells [106]. This excess fluid is routinely drained to alleviate discomfort for patients. Therefore, ascites is a readily available source of ovarian cancer cells for many laboratories. Ovarian CSCs have been isolated from patient ascites and validated via xenograft studies [37]. Although there are not many studies, data suggests the qualitative ‘types’ of CSCs (e.g. ALDH+) are well conserved between ascites and tumor in pair matched patient samples [25]. It must be noted that CSC models developed from ascites may closer model metastatic CSCs than that of the primary tumor. The finding that patient tumor samples preferentially regenerate the malignancy at sub-cutaneous rather than intraperitoneal sites in xenograft mice models supports this concept [32]. As such, ascites derived CSCs models may enable the study of dissemination and propagation of metastatic ovarian cancer. However, such a model may introduce confounding factors if studying recurrent chemoresistance. Fundamentally, the source material for the establishment of CSC models should be defined by the focus of the study.

## A pipeline from clinical intervention to CSC discovery

As we have discussed, a large body of evidence has been generated demonstrating the key role of different populations of CSCs in ovarian cancer. This research must now be driven forward towards a reduction in ovarian cancer mortality rates. This is most likely to be achieved through development of specific ovarian CSC-targeting strategies that complement existing therapeutic regimes. The road to clinical intervention begins with a comprehensive understanding of the specific factors responsible for mortality in specific malignancies (lack of early detection, chemoresistance, metastasis etc.), in line with which appropriate starting material may be chosen. In the case of ovarian cancer, SC/CSC populations from non-malignant, primary, chemoresistant, recurrent and metastatic disease could form a matrix of comparisons. Once identified, CSC populations can be molecularly characterized for mechanisms that maintain SR or drive differentiation. Where a newly-isolated CSC population spontaneously differentiates, comparison of newly-isolated and differentiated samples will facilitate identification of such mechanisms. Once identified, supplementation of the media with the product or inhibitors of these mechanisms has been shown to help maintain SR in culture [107, 108]. Where this is the case, the reversal of this process will activate differentiation, thus providing a stable CSC model system for further analysis.

Once established, stable CSC models can be assayed to identify molecular targets that mark the SR state, which are often useful prognostic and/or diagnostic tools. As CSCs lose their tumorigenic potential upon differentiation, mechanisms that maintain SR and drive differentiation can be targeted as potential therapeutic targets. Importantly, the comparison of CSC from the various aspects of the disease of interest will aid in identification of CSC targets that are less likely to affect the non-malignant SC pool and better affect the specific treatments of patients at different stages of disease.

## Conclusions

To develop CSC-eliminating, cancer curing therapies, stable and malleable ovarian CSC models must first be developed. These models must maintain CSCs in their ‘undifferentiated’ state and must be susceptible to uniform differentiation with the appropriate stimulus. To facilitate the establishment of stable models, CSCs must first be isolated from heterogeneous sources. Analysis of the literature identified CD133+, ALDH+ and HSP+ as the three most robust markers of ovarian CSCs (Table 1). Such isolated CSCs must then be validated as possessing the CSC properties of SR, differentiation and tumorigenesis. We argue that the single cell asymmetric division assay in conjunction with the xenograft tumorigenicity assay is a highly efficient pairing for CSC validation. Stable culture conditions for ovarian CSCs have yet to be established. However, three avenues of investigation are gaining traction. First, spontaneous differentiation of CSCs in culture can be used to identify the pathways involved in SR and differentiation. Second, spheroid growth cultures appear to promote the SR of ovarian CSCs. Full elucidation of the factors involved may result in stable culture conditions for ovarian CSCs. Finally, studies of the CSC niche within tumors can yield insight as to how the ‘undifferentiated’ CSC state is maintained *in vivo* and therefore how stable models may be created *in vitro*. As such, it is likely that stable CSC models will bridge the gap between CSC Discovery and clinical intervention.

## Abbreviations

5-FU: 5-Fluorouracil
ALDH1: Aldehyde dehydrogenase 1
AD: Asymmetric division
CSC: Cancer stem cell
CD: Cluster of Differentiation
EGF: Epithelial growth factor
EMT: Epithelial-mesenchymal transition
EOC: Epithelial ovarian cancer
FGF: Fibroblast growth factor
GSI: γ–secretase Inhibitor
HSC: Hematopoietic stem cell
HSP: Hoechst side population
HLDA: Human Leukocyte differentiation antigen
LIF: Leukemia inhibitory factor
MIS: Müllerian inhibiting substance
NSC: Neural stem cell
SR: Self-renewal
SCF: Stem Cell Factor
SC: Stem cells
TGF-β: Transforming growth factor beta.
